# A Comparative Evaluation of Stress Distribution Around Different Widths of Implant and Bony Interface in Function During Axial and Non-axial Loading: A Finite Element Analysis

**DOI:** 10.7759/cureus.62674

**Published:** 2024-06-19

**Authors:** Deepika R Pai, Damodar M Prabhu, Abdul Sameeh M, Ajay Soman, Anjana Thomas, Anil Varghese Koruthu

**Affiliations:** 1 Department of Prosthodontics, Crown and Bridge, Century International Institute of Dental Sciences and Research Centre, Poinachi, IND; 2 Department of Internal Medicine, W Centre For Preventive Health Care, Mangalore, IND; 3 Department of Prosthodontics, Crown and Bridge, Malabar Dental College and Research Centre, Malappuram, IND; 4 Department of Prosthodontics, Crown and Bridge, Al-Azhar Dental College, Thodupuzha, IND; 5 Department of Prosthodontics, Crown and Bridge, Annoor Dental College and Hospital, Ernakulam, IND; 6 Department of Prosthodontics, Crown and Bridge, Royal Dental College, Palakkad, IND

**Keywords:** implant diameter, finite element analysis, cortical bone, cancellous bone, axial load

## Abstract

Objective: The study employed three-dimensional (3D) finite element analysis (FEA) and examined how implant diameters affect stress distribution across the implant-bone contact and how stress transmission through this interface changes during axial and non-axial loading.

Materials and methods: A 3D mandibular model was created using cone beam CT of a patient with implants inserted into the first mandible molar. Nobel Biocare implants (Nobel Biocare, Switzerland) with specific dimensions of 3.5 mm, 4.3 mm, 5.0 mm, and 6.0 mm were chosen. Models were created in CATIAV5R19 (Dassault Systemes, France) from threaded titanium implant dimensions. Implants were finite element-modeled utilizing ANSYS Workbench v11.0 (Ansys, Inc, Pennsylvania, USA). The analysis involved applying 100 N axial, 50 N buccolingual, and 50 N mesiodistal loads.

Results: In a lower first molar bone segment, the implant top surface was loaded in 100 N axial, 50 N buccolingual, and 50 N mesiodistal orientations. The cortical bone proximal to the implant neck had the most von Mises stress, regardless of model or stress scenario. In Model I cortical bone, maximal stress was centered at the implant neck. Most stress was on lingual bone plates, lesser on buccal, and least on mesial and distal. Less than half of the implant stress was transmitted to the cortical bone. The stress transferred from the implant to the cortical bone in Model II was less than half of the implant stress. The same was true for Models III and IV. In Model I cancellous bone, stress was concentrated in the implant's coronal half and minimal in the apical half.

Conclusion: The stress patterns under axial loading were distributed favorably. Therefore, it can be inferred that an augmentation in the diameter of the implant enhances the even distribution of stress at the interface between the bone and the implant by offering a larger surface area for the dispersion of stress. Furthermore, it was determined that applying force along an implant's axis was a beneficial loading direction and did not negatively impact its lifespan.

## Introduction

The high rate of success and continued monitoring of osseointegrated dental implant patients for over 20 years have fascinated clinicians and researchers globally [1]. Implants might fail throughout the unloaded healing and functional phases for several reasons. Poor dental hygiene, bone quality, patient medical status, and biomechanical variables cause the failure of dental implants. Biomechanical aspects such as loading type, bone-implant contact, implant length and diameter, implant surface form and characteristics, and prosthesis type, along with surrounding bone quantity and quality, have been underlined in the literature [2].

Stress transmission to the bone affects dental implant success. Inappropriate loading stresses the bone surrounding the implant and could lead to resorption. However, low stress may cause disuse atrophy, like the loss of the alveolar bone crest after tooth extraction. Dental implant systems depend on bone-implant biomechanical interaction for long-term functionality. Studies reveal that the tight apposition of bone to the titanium implant is crucial for stress conduction without relative motion or abrasion [3].

The finite element method (FEM) can evaluate and improve the geometry of implants without implantation risk or cost. FEM simulates the intricate structure of the bone-implant interface bond, allowing us to comprehend the loads transferred from the implant to the bone through the interface. It also allows for easy adaptation of the model and visualization of the internal configuration of stress, along with additional mechanical quantities [2]. The study employed three-dimensional (3D) finite element analysis (FEA) and examined how implant diameters affect stress distribution across the implant-bone contact and how stress transmission through this interface changes during axial and non-axial loading.

## Materials and methods

ANSYS Workbench v11.0 (Ansys, Inc, Pennsylvania, USA) was used for the study. Implants were thought to be inserted into the first mandibular molar. The in vivo geometry was closely approximated in the models. The study was broadly divided into two sections: FEM and FEA.

**Figure 1:**
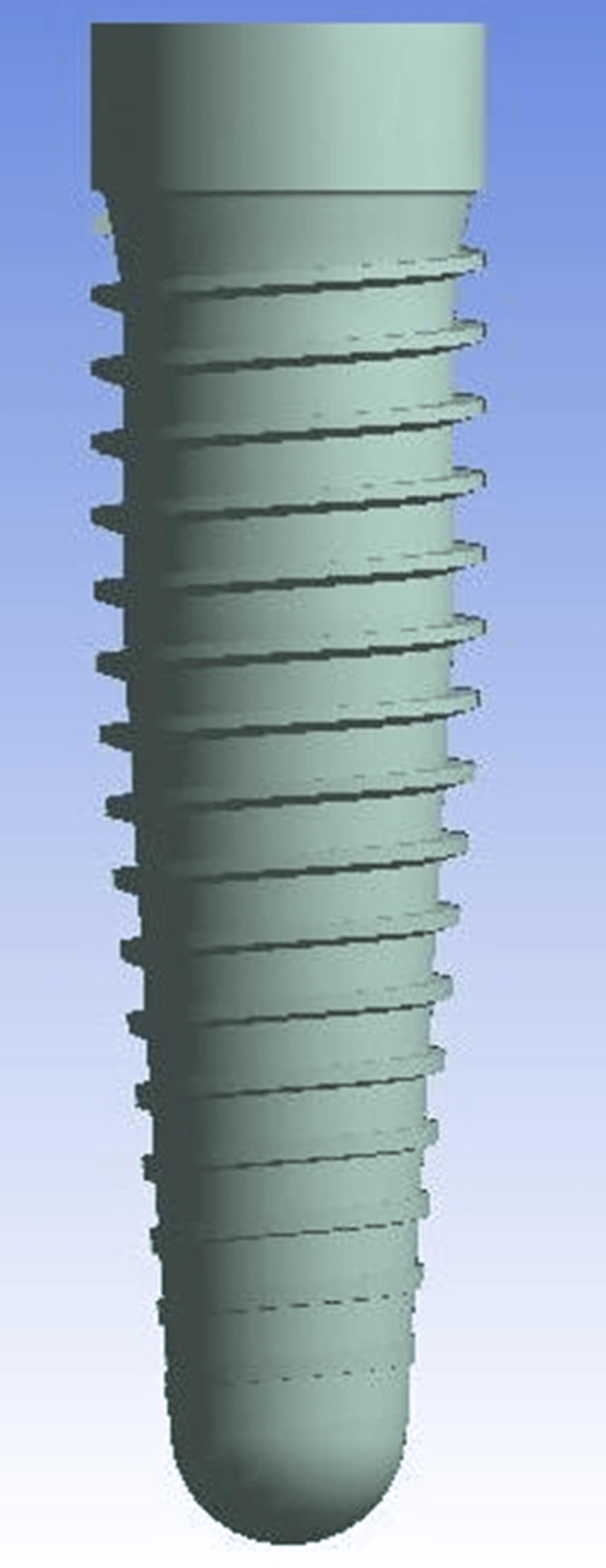
Implant model used for the analysis Image Credit: Author, created using ANSYS Workbench v11.0

**Figure 2:**
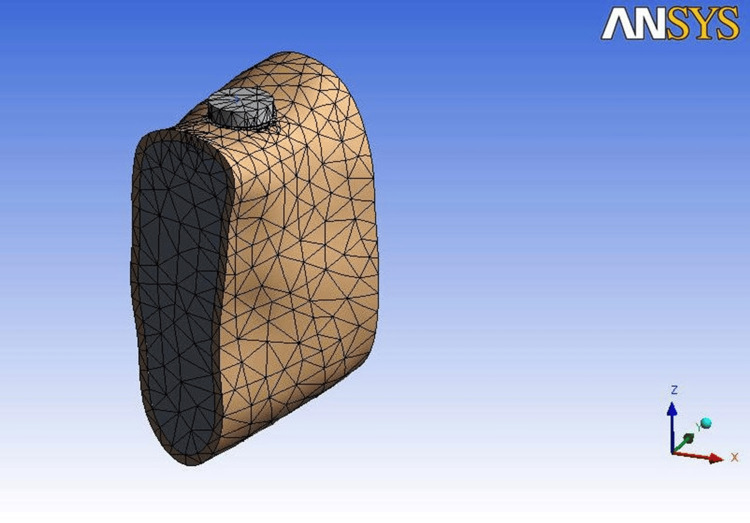
Bone model used for the analysis Image Credit: Author, created using ANSYS Workbench v11.0

**Figure 3:**
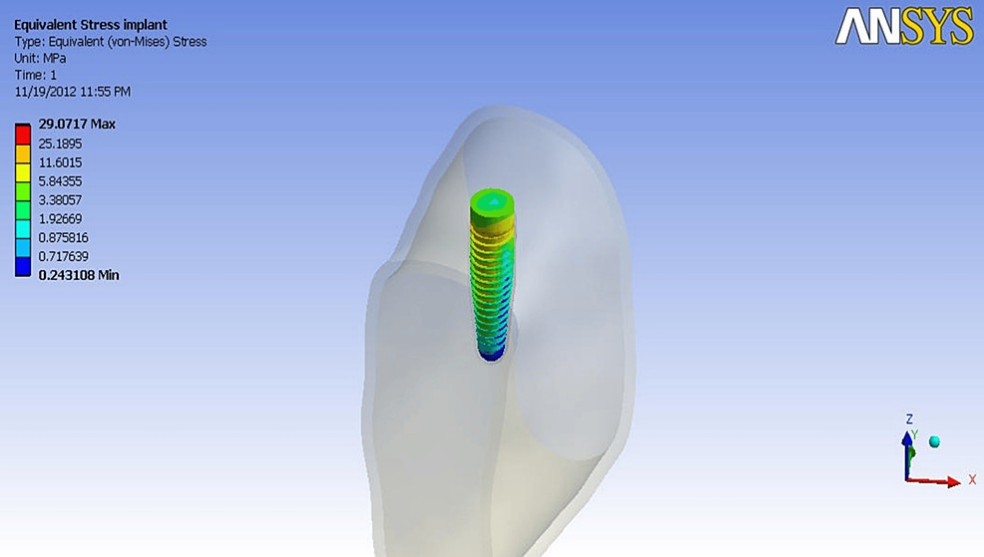
Implant simulated in the bone as the interface Image Credit: Author, created using ANSYS Workbench v11.0

The finite element modeling consisted of geometric model construction, mesh creation, specifying material properties, utilizing boundary conditions, and load application. Geometric model construction was further subdivided into bone modeling, implant modeling, and interface modeling (Figures 1-3).

First, a human mandible CT was obtained [2]. MIMICS-13 (Materialise, Belgium) generated a 3D mandible surface model from 150 axial and 100 coronal CT scans in anteroposterior orientation. Transferring surface models to CATIAV5R19 created 3D solid models. The finalized models were imported into ANSYS Workbench v11.0 in .iges format. Three-dimensional FEM of Nobel Biocare implants was created. The implant dimensions followed product catalog specifications (Table 1) [4].

**Table 1:** Implant dimensions based on the product catalog specifications

Implant diameter (mm)	Collar height	Thread pitch	Major diameter	Minor diameter	Thread height	Overall length	Tip diameter	Collar diameter
3.5	1.5	0.64	3.5	2.96	12.07	13.6	2.11	3.5
4.3	1.5	0.71	4.3	3.67	12.07	13.6	2.56	4.3
5.0	1.5	0.75	5.0	4.18	12.07	13.6	2.98	5.0
6.0	1.5	0.79	5.9	4.97	12.07	13.6	3.54	6.0

The lower grid size prevented the meshing diffusion-bonded microsphere surface, which covered the remaining surface of the implant. Thus, an interface characteristic was derived that integrates cancellous bone and implant surface topography. Models were created in CATIAV5R19 from threaded titanium implant dimensions. Implants were finite element-modeled.

Interface modeling

The implants have solid machined cores and two- to three-layer microsphere threads. On average, microsphere diameter is 100 µm, resulting in a 300 µm thickness. The microsphere dimension was designed so that the thread size formed a bone-ingrowth-friendly gap between the two microspheres. This geometry was unable to be implemented due to the model's reduced grid size; thus, comparable interface features were developed. To replicate bone ingrowth, a row of thin interface components was placed between the implant's threaded surface and the bone. The interface element was believed to be a rectangular cantilever beam of uniform dimension between the implant and bone for the FEM.

Mesh creation

The geometric model's 3D FEM was meshed using ANSYS Workbench v11.0. The default element size (SOLID 187) was used. This study used a 10-noded tetrahedron component with three degrees of freedom per node: x, y, and z translation. The elements were designed for satisfactory solution precision on the size aspect ratio (Table 2).

**Table 2:** Statistics of the implant nodes and elements

Statistics	Implant diameter			
	3.5 mm	4.3 mm	5.0 mm	6.0 mm
Nodes	67707	39305	31322	40538
Elements	37737	20104	16056	22717

Material properties

Young's modulus (Є) and Poisson’s ratio (δ) were used to execute and analyze the program. Cortical, cancellous, and implant bones were assumed to be elastically linear, homogenous, and isotropic. Cortical bone exhibits anisotropic material properties and regional stiffness variations, although it was represented isotropically owing to a lack of data and trouble determining the primary axis. The mechanical characteristics of the interface materials (bone ingrown into a threaded implant surface) were computed analytically as a composite.

The Є of the cortical and cancellous bone used in the model was 13700 MPa, and that of the δ was 0.3. The Є and δ of the titanium alloy were 110000 MPa and 0.35, respectively, and those of the interface were 55750 MPa and 0.35, respectively.

Utilizing boundary conditions

The distal end of the model was constrained in all three directions, and removing the bottom support allowed for bending. These details make the model more clinically accurate.

Applying loads

The applied stresses were within physiologic limits and simulated clinical circumstances. Implants were directly loaded with 100 N axial force. To simplify model fabrication and understanding, the prosthesis was not modeled. The implants were loaded buccolingually with 50 N non-axially. The implants were loaded mesiodistally with 50 N of non-axial force. The analysis was simplified by grouping 12 models.

FEA was performed using ANSYS Workbench v11.0. The processor, or solver, analyzed these models and showed the results in color-coded maps using von Mises stress (VMS) analysis by ANSYS. When VMS exceeds yield strength, metallic implants fail. Thus, they help interpret implant material stresses. The grouping of models based on the direction and magnitude of the forces and diameters of the implants is illustrated in Table 3.

**Table 3:** Grouping of models based on the direction and magnitude of forces and diameters of the implants

Groups	Forces (N)	Directions	Model I (mm)	Model II (mm)	Model III (mm)	Model IV (mm)
Group 1	100	Axial	3.5	4.3	5.0	6.0
Group 2	50	Buccolingual	3.5	4.3	5.0	6.0
Group 3	50	Mesiodistal	3.5	4.3	5.0	6.0

## Results

The implant top surface was loaded in 100 N axial, 50 N buccolingual, and 50 N mesiodistal orientations in a lower first molar bone segment. The cortical bone proximal to the implant neck had the most VMS, regardless of model or stress scenario. In Model I cortical bone, maximal stress was centered at the implant neck. Most stress was on lingual bone plates, lesser on buccal, and least on mesial and distal. Less than half of the implant stress was transmitted to the cortical bone. The stress transferred from the implant to the cortical bone in Model II was less than half of the implant stress. The same was true for Models III and IV. In Model I cancellous bone, stress was concentrated in the implant's coronal half and minimal in the apical half. Table 4 shows cancellous bone VMS values for study models.

**Table 4:** VMS (in MPa) in various diameter implants during axial and non-axial loading VMS: von Mises stress, MPa: megapascal

Diameter (mm)	Loading cases	100 N axial load	50 N buccolingual	50 N mesiodistal
3.5	Implant	24.26	27.282	29.07
	Interface	8.45	10.4	10.944
	Cortical	5.911	10.058	9.7862
	Spongy	1.673	1.0419	0.85338
4.3	Implant	23.763	23.146	26.349
	Interface	6.6136	8.6423	7.4005
	Cortical	5.2334	7.1216	7.1068
	Spongy	1.1604	1.1736	0.7975
5	Implant	22.039	20.931	23.35
	Interface	4.7804	6.381	5.33
	Cortical	4.6907	4.537	6.3024
	Spongy	1.0245	0.725	0.77248
6	Implant	20.872	16.621	19.563
	Interface	3.7407	3.874	3.7147
	Cortical	4.2831	4.029	4.7049
	Spongy	0.9454	0.76607	0.7357

The implant's top surface had 24.26 MPa, the highest stress of any Model I component. The same was true for Models II to IV. As implant sizes increased, interface stress fell dramatically (Models III, IV, and V). Tensile stress was 27.28 MPa on the buccal cortical plate at the implant neck due to buccolingual loading, while compressive stress was mostly at the distolingual cortical bone. The cortical bone was stressed twice as much as when the axial load was applied, which was twice the bucco-lingual load. In Models III and IV, axial and buccolingual loading values remained comparable as implant sizes increased. The greatest VMS in Model I was 29.07 MPa on the mesial and distal sides of the abutment due to mesiodistal loading.

The compressive distal bone plate generated the most stress. The maximum VMS in the mesiodistal direction for Model I in the cortical bone was 9.7862 MPa (Table 4). Mesial tensile stress was 8.569 MPa. Cancellous bone load distribution was similar to cortical bone, except for stress magnitude. Stress was greater at the distal end than at the mesial. Due to mesiodistal loading along the bone segment's long axis, stress was distributed this way. This location had the least stress. Stress levels were similar in the other three models.

The implant neck was stressed mostly in the mesial and distal portions. The mesial side produced tensile tension, and the distal side produced compressive stress. Again, the coronal part of the implant had stress on the mesial and distal surfaces. Stress rarely reaches its apex. Stress dropped from 29 MPa to 19 MPa as implant diameters increased (Models II, III, and IV). Stress was localized on the mesial and distal sides based on load direction. Stress distribution was similar to buccolingual loading in the coronal half of the component. Similar results were seen in Models II, III, and IV.

## Discussion

Implant treatment is predictable, as many clinical studies show success rates above 90% for several implant systems [1]. However, implant failures are mostly caused by biomechanical parameters such as implant and bone mechanical stiffness [5], shape [6], length [7], diameter [8], implant surface topography, direction, and position [9]. Analytical solutions for complex geometries like mandibles are difficult. Therefore, numerical approaches like FEM are needed. For ethical reasons, in vivo strain gauge observations cannot be performed within the bone; hence, FEA measures implant-bone contact stress [10]. This bone section was modeled using a CT scan of a human mandible to accurately approximate cortical and cancellous bone size and shape. The bone segment size was selected to avoid end effects (stress at the bone segment ends) affecting results in the region of interest. A 3D investigation by Sato et al. [8]. found that bone stress around an implant was insignificant if the bone length between the implant and the segment end was at least 4.2 mm. This length was 12.8 mm in this investigation; therefore, the end impacts were minimal and did not change the results.

The limitations at the bone segment end and force application on top of the implant matched only roughly the complicated balance between masticatory forces and how they responded in this investigation. Due to modeling limitations, these simplifications only provide a basic understanding of stress and strain fluctuations under standard circumstances and do not mimic unique clinical scenarios. It was predicted that this simplification would impact the results quantitatively but not qualitatively. Thus, qualitative comparability is preferable to the quantitative information from these analyses [5].

All models showed stress accumulation around the implant neck at the cortical bone level. This supported other findings [2,11,12] from the FEA of loaded implants. Cortical bone's high modulus of elasticity (E=13,700MPa) may increase rigidity and stress resistance. Each of the four models exhibited similar cortical bone stress under vertical loading. Both models considered 100% osseointegration, which may explain it. Clinically, osseointegration is seldom 100% [11]. Studies show that increased osseointegrated surface area improves bone stress distribution. Axial loading caused less implant stress than buccolingual and mesiodistal loading. The load is most effectively supported by the cross-sectional area of the implant due to its alignment with the implant's long axis. The elements under the most stress are around the implant neck; hence, a broader cervical zone may help distribute masticatory pressures. Non-axial loading can cause slight bone loss, osseointegration failure, and implant failure [13]. Therefore, implant-supported prosthesis occlusion planning must be comprehensive.

The FEM is accurate and exact for analyzing structures, yet this study has limitations. First, implant-bone movement was prohibited during loading from multiple angles. Clinically, implants are never 100% osseointegrated [11]. Improved finite element models incorporating dynamic loading circumstances and friction coefficients can be used for further studies [14]. Furthermore, bone-implant interface modeling should include the cortical bone's osseointegration zone of contact and 3D trabecular bone contact patterns [2]. Actual experimental methodologies and clinical trials should adhere to FEA to determine the precise characteristics of the biological system.

## Conclusions

Based on the limitations of this investigation, it can be inferred that the cortical bone experienced the highest level of stress concentration, regardless of the intensity and orientation of the applied load. The stress patterns under axial loading were distributed favorably. Therefore, it can be inferred that an augmentation in the diameter of the implant enhances the even distribution of stress at the interface between the bone and the implant by offering a larger surface area for the dispersion of stress. Furthermore, it was determined that applying force along the axis of an implant was found to be a beneficial loading direction and did not negatively impact its lifespan.
